# Coronavirus disease 2019 (COVID-19) mRNA vaccine effectiveness in asymptomatic healthcare workers

**DOI:** 10.1017/ice.2021.287

**Published:** 2021-06-24

**Authors:** Pablo Knobel, Consol Serra, Santiago Grau, Rocio Ibañez, Pilar Diaz, Olivia Ferrández, Rocío Villar, Alonso Fernando Lopez, Nuria Pujolar, Juan Pablo Horcajada, Marta Roman, Merce Comas, Maria Sala, Xavier Castells

**Affiliations:** 1 Department of Epidemiology and Evaluation, Hospital del Mar Medical Research Institute (IMIM), Barcelona, Spain; 2 Occupational Health Service, Hospital del Mar Medical Research Institute (IMIM), Barcelona, Spain; 3 Department of Pharmacy, Hospital del Mar Medical Research Institute (IMIM), Barcelona, Spain; 4 Hospital del Mar Medical Research Institute (IMIM), Barcelona, Spain; 5 Service of Infectious Diseases, Hospital del Mar Medical Research Institute (IMIM), Barcelona, Spain; 6 Research Network on Health Services in Chronic Diseases (REDISSEC), Barakaldo, Biscay, Spain

The protection offered by the BNT162b2 vaccine (Pfizer–BioNTech) and the mRNA-1273 vaccine (Moderna) to prevent coronavirus disease 2019 (COVID-19) disease has been well documented during phase 3 trials^1,2^ and subsequent observational studies using real-world data.^3^ Healthcare workers (HCWs) have been included in the initial target group to be vaccinated due to their exposure^4^ and their role in transmission^5^ and because they are an essential part of the fight against COVID-19. However, there is little evidence regarding postvaccination severe acute respiratory coronavirus virus 2 (SARS-CoV-2) asymptomatic infection.

Fully understanding the vaccination effect is essential to improving the response to the pandemic within healthcare facilities, and it can also reduce the psychological burden on HCWs. We present the effect of mRNA vaccination on subsequent polymerase chain reaction (PCR) test SARS-CoV-2 positivity in asymptomatic HCWs.

## Methods

This analysis included front-line HCWs of Hospital del Mar in Barcelona, Spain, routinely screened every 2 weeks for SARS-CoV-2 with PCR assays. HCWs were contacted by the occupational health service of the hospital through mobile text messages and had a nasal swab taken by trained personnel. The sample was analyzed in situ in the hospital laboratory. Vaccination began on January 5, 2021. The screening continued throughout and after the vaccination period.

We analyzed 2,462 HCWs screened at Hospital del Mar starting on December 1, 2020, and followed until April 20, 2021 (141 days). We excluded HCWs who had a positive test before December 1. We included only PCR tests performed on asymptomatic HCWs without a known close contact with an infected person within the hospital. Participant age, sex, workplace, and type and dates of vaccine received were obtained from the Hospital administrative database. The mean age of the sample was 38.9 years (SD, 12.4), and 75.5% were female. Participants were unevenly distributed among different types of care units, the most common being in non–COVID-19 wards (44.6%). Most participants were vaccinated with Pfizer–BioNTech (73.5%). In total, 314 HCWs (12.8%) were not vaccinated by April 20, 2021. Although the screenings were periodically scheduled, adherence varied among HCWs: 45.0% had ≥8 tests, 39.0% had 3–8 tests, and 16.0% had 1–2 tests.

## Results

We present the PCR positivity rates grouped by vaccination state. In total, 16.723 PCRs were performed. Test positivity decreased from 1.39% (95% confidence interval [CI], 1.11–1.67) for nonvaccinated HCWs to 0.13% (95% CI, 0.03–0.22) 1 week after the second vaccine dose, resulting in a 90.6% vaccine effectiveness. The PCR tests positivity between 2 weeks after the first dose and 1 week after the second was 0.81% (95% CI, 0.45–1.17), resulting in a 41.7% effectivity (Table 1).

**Table 1.** Percent Positivity According to Vaccination State

Screening Moment	No. of Screenings	No. of Positives	% Positivity	95% CI
Unvaccinated	6,767	94	1.39	1.11–1.67
From vaccination until 14 d after 1st dose	2,076	32	1.54	1.01–2.07
>14 d after first dose until 7 d after 2nd dose	2,350	19	0.81	0.45–1.17
>7 d after second dose	5,530	7	0.13	0.03–0.22

## Discussion

One week after the second dose, vaccination with mRNA vaccines substantially reduced the COVID-19 test positivity and incidence among asymptomatic HCWs. These results are consistent with previous studies regarding mRNA vaccination protection from COVID-19 in healthcare settings.^6^ The protective effect of vaccination 2 weeks after the first dose was weaker than reported in phase 3 trials of the vaccines and other studies conducted in healthcare settings, even when including asymptomatic testing.^7^ The discrepancy might be a consequence of the focus on asymptomatic nonsuspicious cases, which might have gone undetected in previous studies. Previous studies highlight the importance of keeping the guard up in the first days after the first dose of vaccine.^8^ Our findings suggest that vaccine recipients should be aware that the risk of infection is not reduced until at least 1 week after the second dose of vaccine.

Two main limitations of our study should be noted. First, the small number of positives in the vaccinated groups (especially 2 weeks after the second dose) limited our ability to obtain narrower confidence intervals. Second, the rapidly changing dynamics of COVID-19 incidence in the general population might have influenced our results. However, the population incidence remained relatively stable during the study.

Similarly, several strengths should be noted. First, trained professionals gathered the samples, and the samples were analyzed in the hospital laboratory, which ensured high-quality sampling and reduced problems derived from sample handling and transportation. Second, the mandatory proactive screening of asymptomatic HCWs combined with the exclusion of COVID-19 tests to suspected cases among HCWs allowed a very refined view of the vaccine effect on asymptomatic infection. Finally, the follow-up of up to 3 months after the first dose of vaccine allowed us to see the effects beyond the period immediately following vaccination.

Although the results of this study are promising, similar studies should be repeated over time because 2 concerns remain: the effectiveness against rising variants of concern (VoC)^9^ and the period through which the vaccines offer protection.^10^ Both of these factors remain unknown.
